# Natural Language Processing for Substance Use Disorder Information Extraction: A Systematic Literature Review

**DOI:** 10.1007/s40429-026-00733-3

**Published:** 2026-04-11

**Authors:** Ransom J. Wyse, David C. Samuels, Sandra Sanchez-Roige, Lori Schirle, Bethany A. Rhoten, Seo Yoon Lee, Alvin D. Jeffery

**Affiliations:** 1 Department of Biomedical Informatics, Vanderbilt University Medical Center, 2525 West End Ave., Suite 1575, Room 1410B, Nashville, TN 37203 USA; 2 Department of Molecular Physiology and Biophysics, Vanderbilt University School of Medicine, Nashville, TN USA; 3 Department of Psychiatry, University of California San Diego, La Jolla, CA USA; 4 Institute for Genomic Medicine, University of California San Diego, La Jolla, CA USA; 5 Division of Genetic Medicine, Vanderbilt University Medical Center, Nashville, TN USA; 6 Department of Anesthesiology, Vanderbilt University Medical Center, Nashville, TN USA; 7 Vanderbilt University School of Nursing, Nashville, TN USA

**Keywords:** Natural language processing, Systematic review, Substance use disorder, Addiction, Information extraction

## Abstract

**Purpose of Review:**

To examine the use of natural language processing (NLP) for substance use disorder (SUD) information extraction.

**Recent Findings:**

623 studies were reviewed, of which 35 met inclusion criteria. 1 paper (2.9%) was alcohol-related, 12 (34.3%) were opioid-related, 6 (17.1%) were tobacco-related, and 16 (45.7%) included multiple SUDs. Of the three types of NLP categorized for this analysis, 65.7% followed a Rule-Based approach, 37.1% followed a Machine-Learning approach, and 11.4% followed a Deep-Learning approach. NLP methods were categorized into three groups, with 43% as “Most common use” (e.g., concept extraction), 20–35% as “Regular use” (e.g., regular expressions), and < 10% as “Rare use” (e.g., sentiment analysis). Various software applications were used in each included paper, with Python leading (10 papers), followed by cTAKES (9 papers), NegEx (6 papers), R (4 papers) and others. Multiple evaluation metrics were used in each included paper; Multiple SUDs (6 papers) utilized a comparison of F1 scores and ROC AUC, followed by Tobacco (4 papers), Opioids (3 papers), and Alcohol (1 paper), each with acceptable-to-outstanding ROC AUC scores ( > = 0.7) and good-to-excellent F1 scores ( > = 0.7).

**Summary:**

Most papers included in this systematic review encompassed multiple SUDs following Rule-Based approaches, “Most common use” NLP methods (e.g. concept extraction), and familiar software applications (e.g. Python). Evaluation metrics for SUD papers utilizing NLP included common performance metrics, with ROC AUC and F1 scores achieving acceptable-to-outstanding discrimination between classes and good-to-excellent balance between precision and recall, respectively. The future direction of NLP for SUD information extraction could make use of Machine- or Deep-Learning approaches, advanced methods including Regular expressions or Sentiment analysis, and/or advanced software packages designed specifically for NLP endeavors, to better inform public health research and clinical decision making.

## Introduction

Natural language processing (NLP) is the use of machine learning to process and interpret information, such as human language or text data [1]. NLP works through the conversion of data, such as words, into numerical features for further analysis. NLP algorithms include regular expressions, concept extraction, or term-frequency inverse document frequency (TF-IDF). Current research has explored the use of NLP algorithms to characterize substance use disorders (SUD), with many articles using electronic health records (EHR) or unstructured clinical notes as input into NLP algorithms [2].

Substance use disorder, considered both a complex brain disorder and mental illness, has been defined as a chronic, relapsing disorder characterized by compulsive drug seeking and continued use despite harmful consequences, which contribute to long-lasting changes in the brain [3]. The Substance Abuse and Mental Health Services Administration (SAMHSA) adds that these disorders occur when “the recurrent use of alcohol and/or drugs causes clinically significant impairment, including health problems, disability, and failure to meet major responsibilities at work, school, or home” [4]. Epidemiological studies have shown the multifaceted impact of SUDs on both individuals and society. In 2022, 17.3% of people aged 12 or older had a SUD [5]. People with a SUD were associated with a higher risk of suicide mortality [6], and people with schizophrenia were shown to have a significantly higher rate of SUD compared to the general population [7]. Children of parents who have SUDs have been associated with substantial lifetime mental disorders and increased mental burdens. Other societal impacts of people with SUDs include increases in homelessness/poverty, human immunodeficiency virus transmission, criminal behavior, and incarceration [8, 9]. In 2019, the impacts of SUD on both the individual and community amounted to indirect and direct costs of 3.7 trillion dollars in the US [10]. The economic impact of SUD on US hospitals alone in 2017 was more than 13 billion dollars [11].

To date, various preventative measures have been implemented by SAMHSA to combat the impacts of SUD. Such efforts include coordinating interdepartmental agencies, governmental campaigns, and funding outreach areas to raise awareness and organize community resources. The most effective practices have been those that target risk factors for SUD at the individual and community levels to prevent SUD [12]. Moreover, early intervention and detection have also been shown to be most effective in preventing SUD among at-risk population groups, as well as the most cost-effective intervention strategy using screening and brief intervention (SBI) tools [13].

Despite the tremendous personal, societal, and financial burdens SUDs pose, these disorders are treatable and many people (e.g., in some observations, up to 75%) do recover [14, 15]. The use of Screening, Brief Intervention, and Referral to Treatment (SBIRT) tools within hospitals has allowed for an opportunity to engage early within vulnerable populations. Additional screeners include the “Cut,” “Annoyed,” “Guilty,” and “Eye-opener” aid (CAGE-AID) and the Alcohol Use Disorders Identification Test (AUDIT) for alcohol, or the Drug Abuse Screening Test (DAST-10) for other drugs [16]. While these SBIRT screening tools have shown to be effective at identifying SUD and reducing hospital costs, the use of these questionnaires is not yet mainstream or universal in practice. Fragmented or partial use of SBIRT screening tool questions across the clinical landscape as well as patients’ recall bias are among the reasons for hesitancy in acceptance. Given these setbacks, in recent years there has been a paradigm shift in identifying SUD through other means, such as chart review of clinical notes within patients’ EHRs [17].

While NLP has shown to be promising for information extraction, benefiting both chronic disease research (e.g., definitions and prediction modeling) and clinical practice since the early 1990’s [18], this method is relatively nascent as regards SUDs. A systematic literature review, therefore, is essential to determine where the field is currently, and what future direction may be prescient.

## Objectives

The purpose of this systematic literature review is to:

1. Assess peer-reviewed literature to determine how NLP is used for SUD information extraction.
2. For identified SUDs, assess the prevalence of article descriptives, type of NLP, NLP methods, and evaluation metrics utilized.
3. Compare NLP evaluation metrics for SUDs, particularly ROC AUC and F1 scores, to determine performance and discrimination, respectively.

## Materials and methods

A systematic literature review was conducted to investigate the use of NLP techniques to identify information related to individuals with SUDs in the EHR. The articles included in this work followed the Preferred Reporting Items for Systematic Reviews and Meta-Analyses (PRISMA) statement [19]. Results from the search query were uploaded to Covidence systematic review software for manual review by the research team.

## Eligibility Criteria

To be included in the review, an article had to be an indexed peer-reviewed publication of primary research, i.e. authors’ own work, or a peer-reviewed literature review, i.e. authors’ systematic review of the literature at the time of publication. The data in each respective publication must have been sourced from an EHR or electronic medical record (EMR). There were no limits on the publication dates. While substance use disorder is a broad term that encompasses numerous mental health and clinical diagnoses, the phenotypes included in this work were chosen based on SAMHSA’s *Key Substance Use and Mental Health Indicators in the United States: Results from the 2019 National Survey on Drug Use and Health* [20], which included the following: “alcohol,” “amphetamines,” “cannabis,” “marijuana,” “cocaine,” “ecstasy,” “MDMA,” “hash oil(s),” “heroin,” “inhalant,” “LSD,” “methadone,” “opioids,” “phencyclidine,” and “tobacco.” Each term was included by name for search purposes. Further, studies must have described NLP somewhere in their study design to be included; such studies were identified during manual review to identify substance use disorder with a specific NLP algorithm, such as but not limited to regular expressions, concept extraction, dictionary word/phrase matching, transformers, vector embeddings, neural networks, bag of words, or TF-IDF.

This systematic review excluded indexed peer-reviewed publications that failed to meet the inclusion criteria, that were not published in English, that were qualitative and external validation studies, surveys, or animal studies, and that lacked text data, e.g. studies that used NLP only to standardize/structure raw data. Further, studies that could not be reconciled between members of the research team were excluded.

## Data Extraction and Synthesis

For Round 1, a thorough search was conducted in PubMed (NLM), Embase (Ovid), and CINAHL (EBSCOhost) with a publication date prior to March 2024 and no limits on language. A Boolean search strategy was developed with assistance from a health sciences research librarian and used a combination of subject headings, e.g. medical subject headings (MeSH) for articles indexed in PubMed, and relevant keywords to locate literature pertaining to the use of NLP methods or techniques to identify SUD in EHRs. The full database, complete with specific terms and Boolean operators, is shown in Appendix I.

For Round 2, more nuanced abstract and full-text screening was performed using Covidence systematic review software [21]. After removal of duplicate papers, two members of the research team independently extracted the candidate articles and reviewed each title and abstract following the eligibility criteria. Inter-rater agreement between these two reviewers was measured via Cohen’s kappa before a third reviewer, also a member of the research team, reconciled any discrepancies between them. The third reviewer adjudicated any discrepancies by personally reviewing the articles and making a final determination based on the same criteria noted above. At the end of this process, no article remained unreconciled.

In addition to article descriptives including title, year of publication, country of origin, funding source, and conflict of interest disclosures, specific study characteristics were reviewed and recorded for all included studies. For each respective study reviewed, additional variables of interest included the following:

- *Article Descriptives* included type of SUDs described, i.e. the Main SUD Outcome which was categorized as “Alcohol,” “Tobacco,” “Opioids,” or “Multiple”; aims of each study; study eligibility criteria; and study design, e.g. retrospective vs. prospective cohort studies vs. literature reviews.
- *Type of NLP* was a categorical variable created to summarize the NLP algorithms used in each respective paper into one of three distinct groups: (1) “Rule-Based approach,” where the respective authors employed and specifically mentioned manual review of available data or manual rule construction, (2) “Machine-Learning approach,” where the respective authors specifically mention Support Vector Regression/Machines (SVR/M) or use of conditional random fields, and (3) “Deep-Learning approach,” where the respective authors specifically mention the use of transformers, including BERT.
- *NLP Methods* was a categorical variable created to summarize the frequencies of the identified kind of NLP method in each included research paper. NLP methods included, but were not limited to, “Concept extraction,” “Regular expressions,” “Vector embedding,” “Sentiment analysis,” and “Sequence tagging.” For reference, “Speech and Language Processing” is a regularly updated, comprehensive glossary of terms of different NLP methods available from Stanford University that can be accessed online [22].

Arbitrary thresholds for frequency of use were categorized into one of three distinct groups: (1) “Most common use”; (2) “Regular use”; or (3) “Rare use.”

- *Evaluation Metrics*, including the statistical tests used to make comparisons or inferences about model performance, were also included. In studies that utilized multiple evaluation metrics to compare model performance by NLP type, the best performing statistic was examined. In the field of biomedical informatics, particularly for Machine-Learning, an F1 score of 0.5 to 0.7 is considered OK performance, but the model could use improvement. The range of 0.7 to 0.9 is considered good performance for most applications, indicating a good balance between precision and recall, while 0.9 or higher is excellent performance [23]. Similarly, a ROC AUC score of 0.7 to 0.8 is considered good discrimination, 0.8 to 0.9 is considered excellent discrimination, and > 0.9 is considered outstanding discrimination. A score of 1 represents perfect discrimination, in which the model can perfectly distinguish between positive and negative classes [24]. F1 and ROC AUC scores were compared for the studies presented in this systematic review.

All findings are reported as exact summaries from the literature review per the definitions above, and per PRISMA and Covidence.

## Results

### Article Descriptives

A total of 623 studies were imported for screening based on the search query. These included 428 papers from Embase, 145 papers from PubMed, and 50 papers from CINAHL. After removal of 311 duplicate papers, 312 papers were screened for inclusion based on the title and abstract, of which 68 papers were included for full-text screening.

**Fig. 1:**
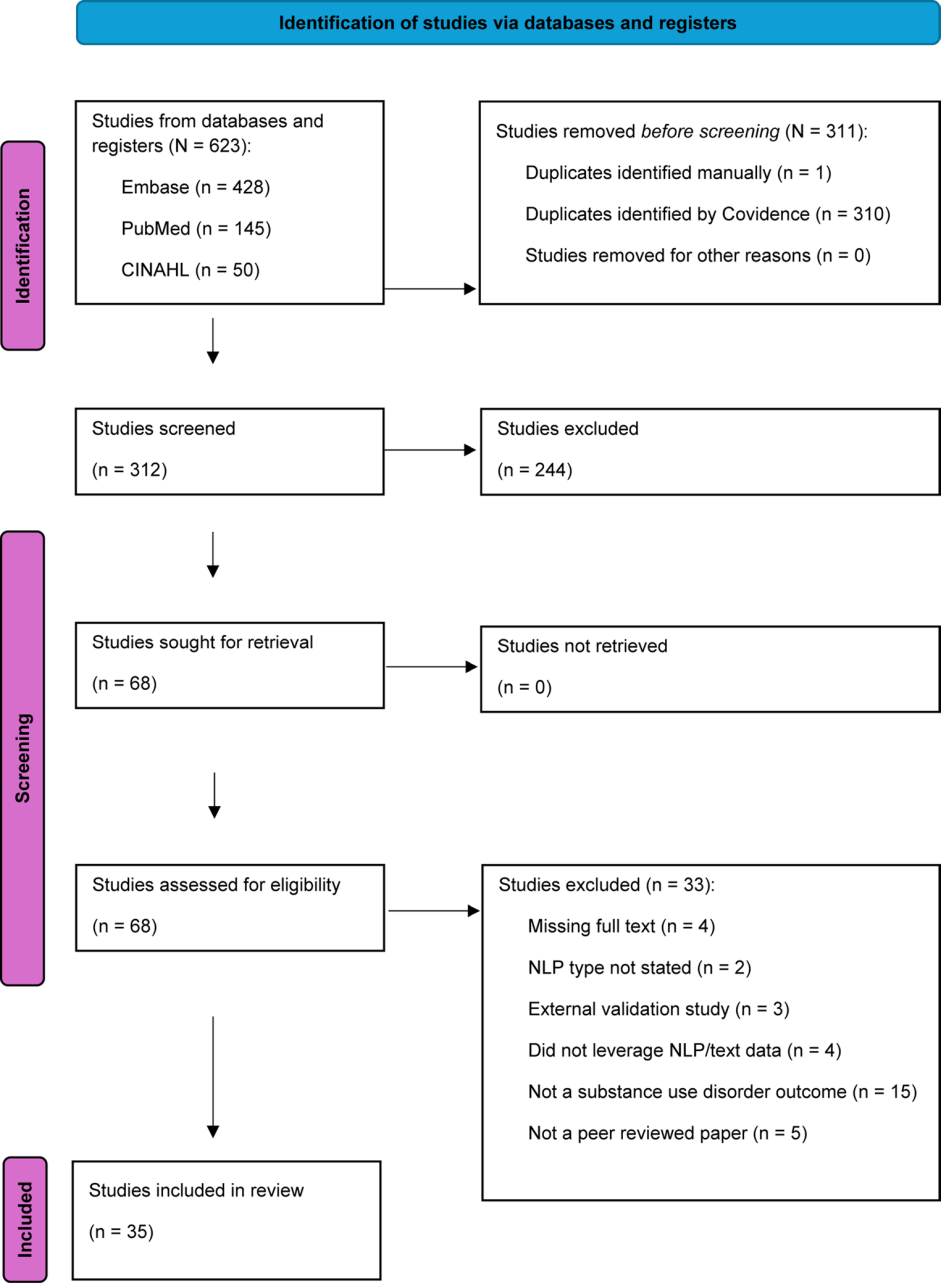
PRISMA Flowchart for Natural Language Processing Systematic Review of Substance Use Disorder Articles

Of the 68 papers assessed for full-text screening, inter-rater agreement was measured with a Cohen’s kappa of 0.51, indicating “moderate agreement.” The two reviewers excluded 15 papers which did not analyze a SUD outcome, 5 which lacked peer-review, 4 which did not leverage NLP or text data, 4 which did not include the full text, 3 which were external validation studies, and 2 which failed to state an NLP algorithm. Once these studies were excluded, 35 studies remained for the systematic review [25–58]. The PRISMA 2020 flow diagram that illustrates these results is shown as Fig. 1.

Almost all (i.e., 32 papers, 91%) papers reported on a US population with the remainder being in the United Kingdom (1 paper), Saudi Arabia and United Kingdom (1 paper), and Sweden (1 paper). Thirty-one papers (89%) reported on hospital-based populations, 3 (8.6%) papers reported on a combination of hospital and community-based populations, and for one paper (2.9%) the population setting could not be determined.

All the papers reviewed listed EHR as a data source. Seven papers (20%) also used an additional data repository, five (14.3%) added data from public databases, and three papers (8.6%) used a combination of EHR data with various combinations of administrative data, public databases and other data repositories.

Papers were categorized into one of four groups, depending on the substance use outcome. Papers that focused exclusively on one type of SUD Outcome, e.g. alcohol dependence, were assigned to that named group. If a particular study mentioned multiple substance use outcomes, e.g. amphetamines, cocaine, and opioids, this was categorized as “Multiple.” Applying this logic, 1 paper (2.9%) was categorized as “Alcohol,” 12 (34.3%) were “Opioid,” 6 (17.1%) were “Tobacco,” and 16 (45.7%) included “Multiple” SUDs.

The earliest study included was published in 2008, and the most recent was published in 2023. Most studies were published between 2019 and 2022 (*n* = 23, 65.7%). Ten (28.6%) included articles were cohort studies, with the majority (*n* = 21, 60%) specifying retrospective cohort studies and 3 (8.6%) specifying prospective cohort studies. One (2.9%) included study was a cross-sectional study. Full details of included studies are presented in Table 1.

**Table 1:** Characteristics of Included Substance Use Disorder Studies for Natural Language Processing Systematic Review

*ID*	*Reference*	*Main SUD Outcome*	*Type of NLP algorithm*	*Specific software or Algorithm used*	*Study design*	*Data Source*
1	Afshar et al., 2022	Multiple: Alcohol, Amphetamines, Cocaine, Ecstasy/MDMA, Opioids	Sentiment analysis; Concept extraction	cTAKES	Retrospective cohort	EHR
2	Joyce et al., 2022	Multiple: Alcohol, Amphetamines, Cocaine, Opioids	Concept extraction	cTAKES	Prospective cohort	EHR
3	Dligach et al., 2019	Multiple: Alcohol, Opioids	Concept extraction; Vector (or word) embeddings/neural networks	cTAKES	Cohort study	EHR; Administrative database; Public data
4	Alzubi et al., 2022	Multiple: Alcohol, Cocaine, Tobacco	Regular expressions; Dictionary-based methods; Negation detection	cTAKES; NegEx; Other: UIMA	Retrospective cohort	EHR; Data repositories
5	Harris et al., 2020	Tobacco	Dictionary-based methods; Negation detection; Concept extraction	NegEx; Other: CLAMP	Cohort study	EHR
6	Ridgway et al., 2021	Multiple: Alcohol, Amphetamines, Cannabis, Cocaine, Methadone, Opioids	Regular expressions; Negation detection	NegEx; Other: Lucene Porter stemmer	Retrospective cohort	EHR; Administrative database; Data repositories
7	Goodman-Meza et al., 2022	Multiple: Amphetamines, Cocaine, Opioids, Benzodiazepine	Regular expressions; Negation detection; N-grams; TF-IDF	NegEx; Other: RegEx	Retrospective cohort	EHR
8	Savova et al., 2008	Tobacco	Negation detection; Bag of words	NegEx; Other: UIMA, Weka	Cohort study	EHR; Public data
9	Bui et al., 2014	Tobacco	Regular expressions	Other: RED algorithm, Smith-Waterman algorithm	Prospective cohort	EHR; Administrative database
10	Lenert et al., 2022	Opioids	N-grams; Vector (or word) embeddings/neural networks; Other: NER	Other: CLAMP	Cohort study	EHR; Data repositories
11	Kashyap et al., 2023	Multiple: Cocaine, Methadone, Opioids	N-grams; Vector (or word) embeddings/neural networks	Other: ClinicalBERT	Retrospective cohort	EHR
12	Yusufov et al., 2022	Multiple: Alcohol, Cocaine, Opioids, Tobacco	Regular expressions	Other: ClinicalRegex Version 1.1.0	Retrospective cohort	EHR
13	Sinha et al., 2017	Opioids	Concept extraction	Other: High-Throughput Phenotyping (HTP)	Retrospective cohort	EHR; Data repositories
14	Zhu et al., 2022	Opioids	Dictionary-based methods; Negation detection; Concept extraction	Other: I2E	Retrospective cohort	EHR; Data repositories
15	Hazlehurst et al., 2019	Opioids	Concept extraction	Other: MediClass	Retrospective cohort	EHR
16	Caccamisi et al., 2020	Tobacco	N-grams; Vector (or word) embeddings/neural networks	Other: Weka	Retrospective cohort	EHR
17	Palmer et al., 2019	Tobacco	Regular expressions	Python	Cohort study	EHR; Public data; Data repositories
18	Hylan et al., 2015	Multiple: Alcohol, Opioids	Regular expressions	Python; cTAKES; Other: SAS	Prospective cohort	EHR
19	Afshar et al., 2019	Alcohol	Concept extraction; TF-IDF	Python; cTAKES; Other: Scikit-learn	Cohort study	EHR
20	Carrell et al., 2015	Opioids	Regular expressions; Dictionary-based methods; Negation detection	Python; NegEx	Retrospective cohort	EHR
21	Ni et al., 2021	Multiple: Alcohol, Cannabis, Opioids, Tobacco	Regular expressions; Concept extraction; Vector (or word) embeddings/neural networks	Python; Other: TensorFlow	Retrospective cohort	EHR
22	Afshar et al., 2019	Opioids	Concept extraction	Python; R; cTAKES	Retrospective cohort	EHR
23	Badger et al., 2019	Opioids	Dictionary-based methods; Concept extraction	Python; R; cTAKES	Retrospective cohort	EHR
24	Sharma et al., 2020	Opioids	Dictionary-based methods; Concept extraction; N-grams; TF-IDF; Vector (or word) embeddings/neural networks	Python; R; cTAKES	Cohort study	EHR; Data repositories
25	Schirle et al., 2021	Opioids	Concept extraction; TF-IDF	Python; R; spaCy	Retrospective cohort	EHR; Data repositories
26	Singleton et al., 2023	Opioids	Regular expressions; Dictionary-based methods	Python; spaCy; Other: Natural Language Toolkit (NLTK)	Retrospective cohort	EHR
27	Rajendran et al., 2020	Tobacco	Concept extraction; TF-IDF; Vector (or word) embeddings/neural networks	Python; word2vec; Other: Keras, Gensim, Noble Coder	Retrospective cohort	EHR; Data repositories
28	Irving et al., 2021	Multiple: Amphetamines, Cannabis, Cocaine, Ecstasy/MDMA	Dictionary-based methods	R	Retrospective cohort	EHR
29	Topaz et al., 2019	Multiple: Alcohol, Cocaine, Opioids	Negation detection; Vector (or word) embeddings/neural networks	R; word2vec; Other: NimbleMiner, phrase3vec	Cohort study	EHR; Public data
30	Mitra et al., 2021	Multiple: Alcohol, Opioids, Tobacco	Vector (or word) embeddings/neural networks	spaCy; Other: BERT	Other: cross-sectional study	EHR; Public data
31	Poulsen et al., 2022	Opioids	Sentiment analysis; Concept extraction	spaCy; Other: EMPATH, ConText	Cohort study	EHR; Public data
32	Lybarger et al., 2023	Multiple: Alcohol, Cocaine, Opioids, Tobacco	Dictionary-based methods; N-grams; TF-IDF; Vector (or word) embeddings/neural networks; Other: retrained language models (LM), sequence tagging	word2vec; Other: BERT, T5	Cohort study	EHR; Public data
33	Lingeman et al., 2017	Opioids	Sentiment analysis; N-grams; Vector (or word) embeddings/neural networks	word2vec; Other: NLTK tokenizer, SentiWordNet	Retrospective cohort	EHR
34	Feller et al., 2020	Multiple: Alcohol, Amphetamines, Cannabis, Cocaine, Opioids	Bag of words; TF-IDF	Not specified	Retrospective cohort	EHR
35	Haller et al., 2017	Multiple: Alcohol, Cannabis, Opioids	Regular expressions; Negation detection	Not specified	Retrospective cohort	EHR

EHR: Electronic Health Record

### Type of NLP

A total of 23 (65.7%) papers utilized a “Rule-Based approach,” which included manual review of available data or manual rule construction, followed by 13 (37.1%) utilizing a “Machine-Learning approach,” which included SVR/M or use of conditional random fields, and lastly 4 (11.4%) utilized a “Deep-Learning approach,” where the respective authors specifically mention the use of transformers, including BERT (Table 2).

**Table 2:** Natural Language Processing Approach, Stratified by Substance Use Disorder Outcome

SUD Article Type	Rule-Based Approach: *n* (%)	Machine-Learning Approach: *n* (%)	Deep-Learning Approach: *n* (%)
Alcohol Use	0 (0%)	1 (2.9%)	0 (0%)
Opioid Use	9 (25.7%)	4 (11.4%)	1 (2.9%)
Tobacco Use	2 (5.7%)	5 (14.3%)	0 (0%)
Multiple	12 (34.3%)	3 (8.6%)	3 (8.6%)
Total	23 (65.7%)	13 (37.1%)	4 (11.4%)

## NLP Methods

**Fig. 2:**
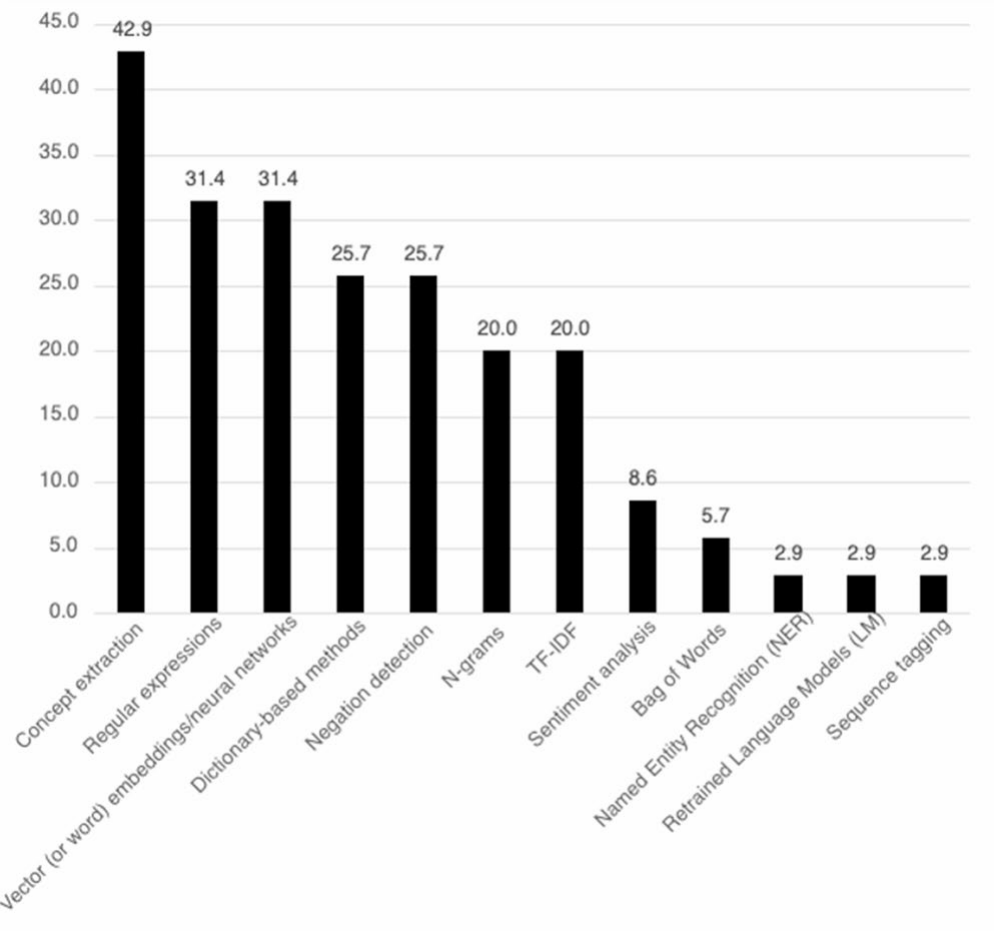
Proportion of Articles Containing Specific Natural Language Processing Methods in Substance Use Disorder Systematic Review

The NLP methods employed in these papers can be broken into three groups based on their frequency of use. Concept extraction, which was employed in 43% of papers, was the most-utilized NLP method and therefore was categorized as “Most common use.” Regular expressions, Vector embedding, Dictionary-based methods, Negation detection, TF-IDF and N-gram methods, were used in 20–35% of the papers, which were categorized as “Regular use.” Finally, less than 10% of papers utilized Sentiment analysis, Bag of Words, Retrained Language Models, Sequence tagging and Named Entity Recognition (NER), which were categorized as “Rare use.” Twenty-eight (80%) papers employed multiple NLP methods. The proportional use of each method is illustrated in Fig. 2.

## NLP Software Applications

**Fig. 3:**
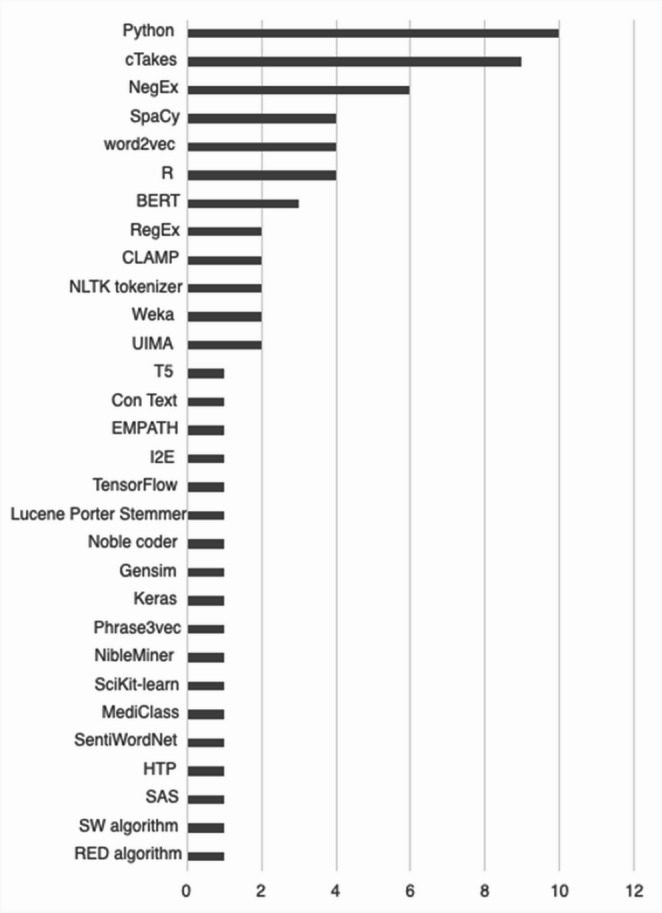
Frequency of Software Applications Used in Substance Use Disorder Articles

A broad range of software was used to implement the NLP analyses among the eligible articles (Fig. 3). Python was widely used. The highest-ranking NLP-specific software was Clinical Text Analysis and Knowledge Extraction System (cTAKEs), which was used in nine papers (25.7%). NegEx, a tool for detecting negation in text, was used in six papers (17.1%). SpaCy, a python open-source library for NLP, was used in four papers (11.4%) and Word2vec was used in four papers (11.4%).

### Evaluation Metrics

Most of the studies investigating “Multiple” SUDs utilized F1 scores (*n* = 4) and ROC AUC (*n* = 3), followed by only F1 scores for “Tobacco” (*n* = 4), F1 scores (*n* = 3) and ROC AUC (*n* = 2) for “Opioids,” and only 1 ROC AUC score for “Alcohol.” For each score identified in our analysis, a color code was assigned to denote model performance, with gray indicating “OK” F1 score performance and “good discrimination” for ROC AUC, orange denoting “good” F1 score and “excellent discrimination” for ROC AUC, and green denoting “excellent” F1 score and “outstanding discrimination” for ROC AUC.

F1 and ROC AUC evaluation metrics by SUD article type are illustrated in Table 3 below. The alcohol paper achieved the lowest evaluation score, with an AUC ROC of 0.78, denoted as the gray color. Opioid, Tobacco, and Multiple SUD papers each achieved F1 scores ranging from 0.78 to 0.99, indicating good-to-excellent performance, denoted as orange and green, respectively. Similarly, ROC AUC scores ranged from 0.89 to 0.96 for these papers, indicating excellent-to-outstanding discrimination, denoted as orange and green, respectively.

**Table 3:**
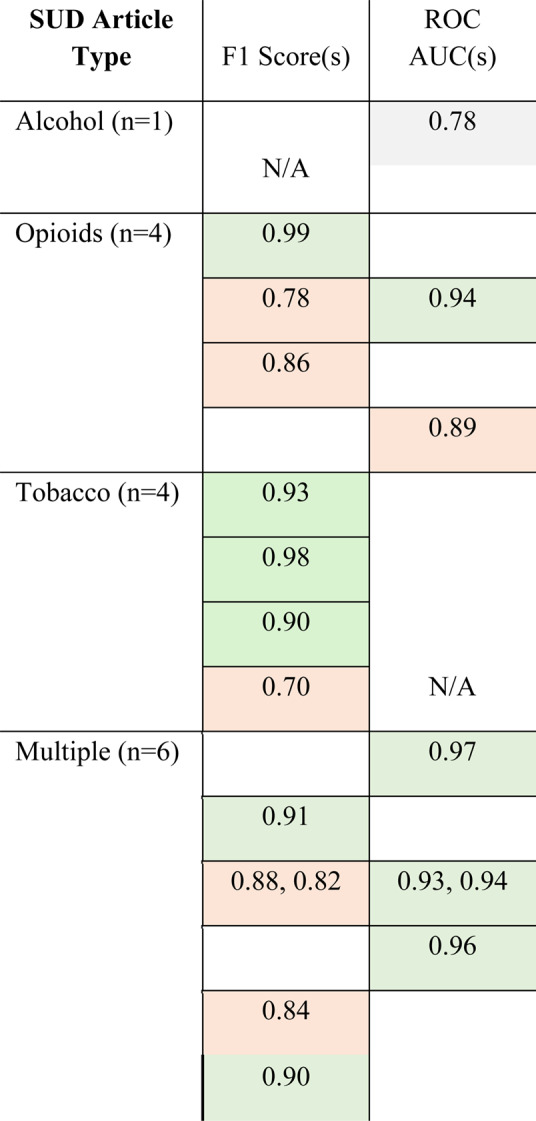
NLP Systematic Review F1 and ROC AUC Evaluation Metrics, Stratified by SUD Article Type

## Conclusion

This systematic review of SUD literature sought to identify the use of NLP in extracting information to characterize SUDs from varied data sources.

Most papers meeting eligibility criteria for this systematic review encompassed Multiple SUDs (*n* = 18) following Rule-Based approaches, “Most common use” NLP methods (e.g. Concept extraction), and familiar software applications (e.g. Python). Given its importance to society and research alike, alcohol, surprisingly, encompassed the fewest number of articles as a featured substance (*n* = 1), however, it was included in nearly 3/4 of studies investigating multiple SUDs, i.e., 13/18 (72.2%). Evaluation metrics for the included SUD papers utilizing NLP included common performance metrics, with ROC AUC and F1 scores achieving acceptable-to-outstanding discrimination between classes and good-to-excellent balance between precision and recall, respectively.

This systematic review noted a few important similarities between SUD articles. First, there was a rapid increase in papers related to SUDs beginning in 2018 in the US, with a low number of studies on cohorts in the international setting. These papers employed a low utilization of certain NLP methods, such as Bag of Words, Retrained Language Models, Sequence tagging and NER. We believe these NLP methods may indicate future areas of development in research application, including the use of TF-IDF for phenotype extraction in an unstructured data source, such as an EHR. Second, there was a lack of broad use of NLP-specific software with the notable exception of cTAKES; no other NLP tool had wide usage. Researchers, it appears, are still writing their own NLP software in R and Python for SUD-related analyses. Third, evaluation metrics for SUD papers utilizing NLP included common performance metrics, with ROC AUC and F1 scores achieving acceptable-to-outstanding discrimination between classes and good-to-excellent balance between precision and recall, respectively.

NLP for SUD information extraction can better inform public health research and clinical decision-making in multiple ways. While still nascent as regards its application to SUDs, Singleton et al. have underscored the import of NLP methods for opioid research specifically, namely from Rule-Based approaches. They argue that NLP is advantageous in terms of “improving the completeness of ascertainment,” and “mitigating biases in EHR structured data quality with regard to the age, gender, and race/ethnicity of the patient.” Further, they argue that many diseases studied using NLP methods have been non-SUD related, such as cancer, venous thromboembolism, peripheral arterial disease, and diabetes mellitus [59]. Their work showed that the limited use of NLP in SUD research had previously involved specific, vulnerable populations, such as patients undergoing chronic opioid therapy. In their attempts to expand the scope, the authors demonstrated that when NLP for opioid use disorder information extraction was applied to a more generalizable population of hospital patients, surveillance improved as compared to more traditional research methods, like those that only rely on ICD-10-CM codes [59]. These findings parallel the emerging trends we note for Opioids and SUDs in general in this systematic review, in that a growing number of SUD researchers seem to be applying Rule-Based approaches to databases such as EHR. The implications are that using NLP methods to improve surveillance in a generalizable population data source, like EHR, would improve epidemiological studies. Clinical decision-making also can take advantage of NLP for SUD information extraction. In their respective studies, Sato et al. and Goodman-Meza et al. found that NLP was effective in identifying hospitalizations of people who use drugs [60] and people who inject drugs [61], surpassing traditional methods of identification including manual chart review. Moreover, compared to manual chart reviews, NLP information extraction has the added benefit of being blind to inherent biases, such as inherent clinician biases toward patients of low income or those from racially and ethnically minoritized populations [62]. In a clinical field involving SUD, this bias is noteworthy and must be mitigated if not eliminated; NLP information extraction may improve the effect of this bias by automating data collection and improving case identification.

This systematic review is not without limitations. First, there may be selection and reporting biases regarding the included studies; those articles with statistically significant findings may be flooding the SUD literature, as is evident with the number of significant F1 and AUC ROC scores in this corpus. However, it is noteworthy to mention that 15/35 (42.9%) papers included in this review utilized these metrics, with the remainder comprising other metrics such as confidence intervals, Kappa statistics, or regression methods. Second, this study critically assessed the importance of NLP types and methods, without properly assessing the availability of such tools to the respective research teams; for example, a particular research team may have selected a common NLP approach with common NLP software to address their research question due to the convenience or availability of such sources, whereas a more complicated/expensive approach or software package may have been out of reach but otherwise preferred.

The importance of identifying data extraction methods for common SUDs in literature speaks to the significance of emerging research in a complicated field of study. Identifying common themes in data extraction techniques used *today* addresses the approach to research and clinical decision-making *tomorrow*. The findings of this systematic review reveal how NLP information extraction for SUDs offers insight into streamlining research practice via its application to generalizable data sources like EHRs, without bias, and as an alternative to traditional practices such as heavy reliance on chart reviews for data extraction, and administrative codes like ICD-10-CM for case definitions. From a clinical perspective, the use of NLP information extraction for SUDs showcases how to better serve this vulnerable population via unbiased screening at all documented points along the patient journey. The future direction of NLP information extraction for SUDs could make use of Machine- or Deep-Learning approaches, advanced methods including Regular expressions or Sentiment analysis, and/or advanced software packages designed specifically for NLP endeavors, to further improve surveillance and reduce bias.

## Data Availability

No datasets were generated or analysed during the current study.

## Appendix I. Full Search Strategies Utilized for Systematic Review

**PubMed (NLM)**

Number of results: 145 

**Table:**

Search	Query
1	alcohol[tiab] OR amphetamine*[tiab] OR cannabis[tiab] OR chemical[tiab] OR cocaine[tiab] OR drug*[tiab] OR ecstasy[tiab] OR “hash oil“[tiab] OR hashish[tiab] OR heroin[tiab] OR inhalant*[tiab] OR lsd[tiab] OR marihuana[tiab] OR marijuana[tiab] OR mdma[tiab] OR methadone[tiab] OR methamphetamine*[tiab] OR morphine[tiab] OR narcotic*[tiab] OR opiate*[tiab] OR opioid*[tiab] OR opium[tiab] OR phencyclidine*[tiab] OR polydrug[tiab] OR substance[tiab] OR tobacco*[tiab]
2	“illicit drugs“[mesh] OR “alcohol drinking”[mesh] OR “amphetamines”[mesh] OR “cannabis”[mesh] OR “cocaine”[mesh] OR “designer drugs”[mesh] OR “heroin”[mesh] OR “methamphetamine”[mesh] OR “narcotics”[mesh]
3	1 OR 2
4	abstain*[tiab] OR abstin*[tiab] OR abus*[tiab] OR addict*[tiab] OR consum*[tiab] OR dependen*[tiab] OR disorder*[tiab] OR excess*[tiab] OR habituation[tiab] OR misuse[tiab] OR overdose[tiab] OR problem*[tiab] OR risk*[tiab] OR withdrawal*[tiab]
5	3 AND 4
6	“Substance-Related Disorders“[Mesh]
7	5 OR 6
8	“natural language processing”[tiab] OR “regular expression”[tiab] OR “regular expressions”[tiab] OR transformer*[tiab]
9	“natural language processing“[mesh]
10	8 OR 9
11	ehr[tiab] OR Electronic Health Record[tiab] OR Electronic Health Records[tiab] OR Electronic Medical Records[tiab] OR Electronic Medical Record[tiab] OR Computerized Medical Record[tiab] OR Computerized Medical Records[tiab]
12	“Electronic Health Records“[Mesh]
13	11 OR 12
14	7 AND 10 AND 13

**CINAHL (EBSCOhost)**

Number of results: 50

(((alcohol OR amphetamine* OR cannabis OR chemical OR cocaine OR drug* OR ecstasy OR hash oil OR hashish OR heroin OR inhalant* OR lsd OR marihuana OR marijuana OR mdma OR methadone OR methamphetamine* OR morphine OR narcotic* OR opiate* OR opioid* OR opium OR phencyclidine* OR polydrug OR substance OR tobacco* OR (MH “Street Drugs+”) OR (MH “Alcohol Drinking+”) OR (MH “Amphetamines+”) OR (MH “Cannabis+”) OR (MH “Cocaine+”) OR (MH “Designer Drugs”) OR (MH “Heroin”) OR (MH “Methamphetamine+”) OR (MH “Narcotics+”)) AND (abstain* OR abstin* OR abus* OR addict* OR consum* OR dependen* OR disorder* OR excess* OR habituation OR misuse OR overdose OR problem* OR risk* OR withdrawal*)) OR (MH “Substance Use Disorders+”)) AND (“natural language processing” OR “regular expression” OR “regular expressions” OR transformer* OR (MH “Natural Language Processing”)) AND (ehr OR “Electronic Health Record” OR “Electronic Health Records” OR “Electronic Medical Records” OR “Electronic Medical Record” OR “Computerized Medical Record” OR “Computerized Medical Records” OR (MH “Electronic Health Records+”)).

**EMBASE (OVIDsp)**

Limited to article or article in press or “review”.

Number of results: 428

(((alcohol or amphetamine* or cannabis or chemical or cocaine or drug* or ecstasy or hash oil or hashish or heroin or inhalant* or lsd or marihuana or marijuana or mdma or methadone or methamphetamine* or morphine or narcotic* or opiate* or opioid* or opium or phencyclidine* or polydrug or substance or tobacco* or exp illicit drug/or exp drinking behavior/or exp amphetamine derivative/or exp cannabis addiction/or exp cannabis/or exp cocaine/or exp cocaine dependence/or exp designer drug/or exp diamorphine/or exp methamphetamine/or exp narcotic agent/) and (abstain* or abstin* or abus* or addict* or consum* or dependen* or disorder* or excess* or habituation or misuse or overdose or problem* or withdrawal*)) or exp drug dependence/) and (natural language processing or regular expression or regular expressions or transformer* or exp natural language processing/) and (ehr or electronic health record or electronic health records or electronic medical records or electronic medical record or computerized medical record or computerized medical records or exp electronic health record/).
